# Microwave-Assisted Formation of Ternary Inclusion Complex of Pterostilbene

**DOI:** 10.3390/ph16121641

**Published:** 2023-11-22

**Authors:** Yousef A. Bin Jardan, Abdul Ahad, Mohammad Raish, Abdullah M. Al-Mohizea, Fahad I. Al-Jenoobi

**Affiliations:** Department of Pharmaceutics, College of Pharmacy, King Saud University, Riyadh 11451, Saudi Arabia

**Keywords:** ABTS, antioxidant, dissolution, DPPH, inclusion complex, pterostilbene, solubility, ternary inclusion complex, β-cyclodextrin

## Abstract

Pterostilbene (PTS) is a naturally occurring phytoalexin. PTS displays limited water solubility, which consequently results in its diminished oral bioavailability. Therefore, a ternary inclusion complex (TIC) of PTS with β-cyclodextrin (βCD) in the presence of ternary substance Pluronic^®^ F-127 (PLF) was prepared using microwave technology. The PTS-TIC was characterized by dissolution performance. Further, the prepared TIC was characterized by DSC, FTIR, NMR, XRD, and SEM analysis. Additionally, the antioxidant activity of PTS and PTS-TIC was also evaluated. Phase-solubility studies revealed that PTS’s solubility in water was increased by 6.72 times when βCD/PLF was present. In comparison with PTS, prepared PTS-TIC produced a considerable improvement in PTS release. After 1 h, 74.03 ± 4.47% of PTS was released from PTS-TIC. Outcomes of DSC, FTIR, NMR, XRD, and SEM analysis revealed that the PTS was enclosed in the βCD cavity. In terms of antioxidant properties, the PTS-TIC formulation demonstrated superior activity compared to PTS, possibly attributed to the improved solubility of PTS resulting from the formation of TIC using microwave technology. It was concluded that microwave technology proved to be an extremely beneficial means of interacting PTS with βCD. In addition to increasing the solubility of PTS, the findings are also expected to improve its bioavailability by increasing its solubility. As a result, this study could provide insight into potential methods for enhancing the solubility of polyphenolic substances like PTS.

## 1. Introduction

**Figure 1:**
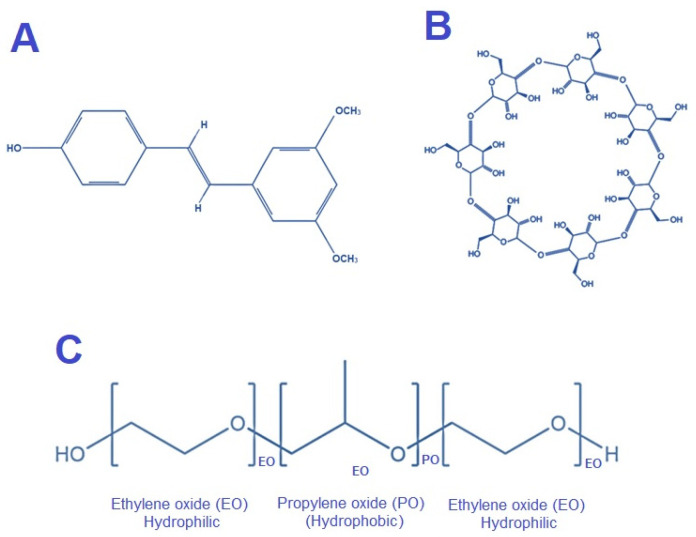
Chemical structure of (**A**) PTS, (**B**) βCD, and (**C**) PLF.

Pterostilbene (Figure 1A) is a naturally occurring phytoalexin. It is part of a group of phenolic compounds called stilbenes [1,2]. The promising therapeutic potentials attributed to pterostilbene have resulted in an increase in its consumption in a wide range of foods containing stilbene. There are some types of grapes, blueberries, and wines that fall into this category [3]. In addition, a number of medications contain it as well. There are a number of biological activities associated with its pharmacological properties, such as analgesic, anticancer, anticholesterol, antifungal, antihyperglycemic, anti-inflammatory, antioxidative, and hypolipidemic activities [1,4,5]. However, pterostilbene possesses poor physicochemical properties, is often oxidized by a number of enzymes, such as laccase, and exhibits poor solubility in water and bioavailability [6]. A variety of approaches have been explored over the years with the goal of improving solubility, including cyclodextrin (CD)-drug complexation [7], salts and co-crystals of pharmaceuticals [8], chemical modifications [9], lipid-based formulations [10], polymorphic modifications [11] cosolvents [12], biopolymers [13], and micelles [14]. The CDs are of natural origin and are currently widely used in pharmaceuticals as carriers for several poorly water-soluble drug(s) and are expected to remain popular for many years to come [15]. PTS is placed in category II of the Biopharmaceutical classification system [1,16]. Therefore, pterostilbene should be complexed with molecules such as CDs that increase its bioavailability, solubility, and stability. βCD is considered a ‘natural product’ in Japan and ‘generally recognized as safe’ (GRAS) in the United States [17].

In addition to increasing the solubility of several poorly water-soluble drugs, CDs accelerate their dissolution rate [18]. In CDs, glucose units are attached to α-(1, 4) oligosaccharide to produce a torus-shaped structure. There are three types of CDs that are most commonly used, namely, αCD, βCD (Figure 1B), and γCD, each containing 6, 7, and 8 glucose units correspondingly [19,20]. The concentric configuration of α-glucose in which primary hydroxyl groups are present on one face and secondary hydroxyl groups at the opposite face gives rise to a hollow truncated cone shape structure. The existence of dynamical flip-flop hydrogen bonds in the secondary hydroxyl rim (O2–H2···H3–O3) of βCD makes the structure rigid and is responsible for the low solubility [21]. Moreover, the central cavity is more hydrophobic than either face; hence, small hydrophobic molecules can comfortably be encapsulated in the cavity [22,23,24]. Noncovalent interactions between compounds and the hydrophobic moiety of amphiphilic molecules with CDs form inclusion complexes, which have remarkable water solubility [25]. Nevertheless, a number of variables, including the type of CD, can affect the solubility of such inclusion complexes [15,26,27]. In particular, βCD has been thoroughly investigated for its potential make inclusion complexes for several classes of inadequately water-soluble drugs in order to facilitate their water solubility [28]. βCD having a cone-shaped structure in which a wide range of hydrophobic guest substances can be enclosed in its non-polar pocket of βCD [29,30]. Several studies indicate that the use of a ternary system having a drug, CD, and a ternary substance improves the efficiency of complexation and helps to decrease the CD content [31]. A wide variety of ternary substances have been investigated for the formulation of inclusion complexes containing poorly water-soluble drug(s) [32,33]. Shah et al. prepared cefuroxime axetil inclusion complexes with HPβCD and a variety of ternary substances, including PVP K30, poloxamer, PEG 400, and HPMC. The authors concluded that, in contrast to binary inclusion complexes, ternary inclusion complexes have a much higher stability constant [34]. A ternary inclusion complex (TIC) of piperine has also been developed by Alshehri et al. Incorporating hydroxypropyl methylcellulose and CD. As compared with pure piperine or binary inclusion complex, the ternary inclusion complex enhanced piperine’s solubility and dissolution [35]. In another finding, authors developed asiaticoside inclusion complexes using CD, chitosan, and poloxamer. It was noted that, as compared to binary complexes, ternary complexes exhibit higher permeation, solubility, and dissolution rates [36]. According to several studies, water-soluble polymers can be incorporated into ternary complexes to increase drug solubility [37,38,39,40,41]. Additionally, CDs have been demonstrated to amplify the antioxidant activities of a wide range of compounds by increasing the rate at which they are released from inclusion complexes [42,43]. The FDA approved Poloxamer for use in pharmaceutical products, a linear non-ionic polymer that is non-toxic and does not cause allergic reactions. It consists of two hydrophilic parts that are connected to the lipophilic parts. A common application for it is to enhance solubility of poorly soluble drugs due to its surfactant properties [44,45]. Further, the traditional methods of inclusion complex formulation mainly involve kneading, physical mixing, and spray drying technologies. While the freeze-drying and co-precipitation processes are capable of producing inclusion complexes, albeit there are certain disadvantages associated with them. A major disadvantage of these methods is the lengthy processing time [46]. On the other hand, microwave-assisted preparation of inclusion complex technique is becoming increasingly popular in recent times owing to the fact that it involves very little time involved in the reaction as well as it being more environmentally friendly since it makes use of minimal solvents, lowering the reaction temperature and reducing by-product quantities [47]. This investigation explored the effects of the TIC of βCD/Pluronic F127 (PLF, Figure 1C) on PTS’s aqueous solubility and dissolution rate. A TIC of PTS and βCD + PLF was prepared using microwave irradiation. The apparent stability constant (Ks), an indicator of the attraction of the PTS to the CD in water and complexation efficiency (Ce), was determined through phase solubility studies. Furthermore, the dissolution characteristics of the ternary physical mixture (TPM) and PTS-TIC were compared to PTS alone. Furthermore, PTS, βCD, PLF, and PTS-TIC were examined for solid-state characterization. As well, the antioxidant activity of prepared PTS-TIC was determined by DPPH and ABTS assays.

## 2. Results and Discussion

### 2.1. Phase Solubility

**Figure 2:**
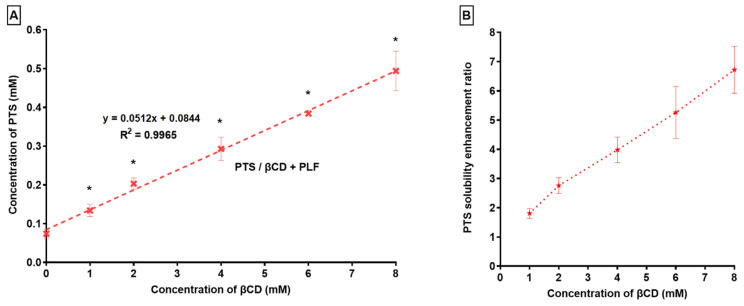
(**A**) PTS Phase solubility and (**B**) PTS solubility enhancement ratio in the context of βCD and PLF. “* *p* < 0.05 in comparison with *S*_0_” (*n* = 3, Mean ± SD).

Phase solubility analysis has been conducted on PTS’s solubility in βCD with PLF. As shown in Figure 2, an increase in the content of βCD led to an enhancement in the solubility of PTS.

It was determined that PTS’s solubility in water increased 6.72 times when βCD/PLF was present. Research indicates that the ternary substance (PLF) establishes a connection with the CD surface, in addition to PTS-CD, thereby facilitating the production of co-complexes [48,49]. The *Ks* and *Ce* values were 649 ± 112 M^−1^ and 48 ± 8%, respectively. According to previous findings, Ks values of 50 to 5000 M^−1^ were most appropriate for enhancing poorly water-soluble drugs’ stability and solubility [50,51,52,53,54,55]. Based on our analysis, the *Ks* value was 649 ± 112 M^−1^, which indicated that the PTS/βCD/PLF complex was stable [56]. It is evident from the results that substantial interactions among PTS and βCD/PLF have been accomplished, as demonstrated by the higher Ks value.

### 2.2. Dissolution Study

**Figure 3:**
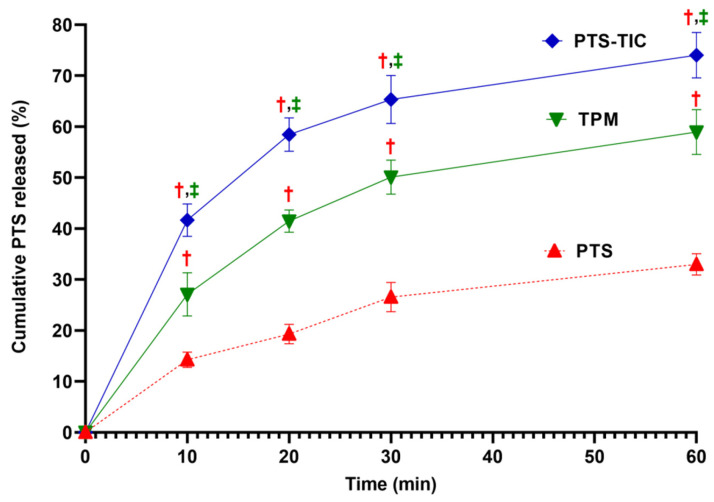
Illustration showing an overview of dissolution of PTS, TPM, and PTS-TIC, “^†^ *p* < 0.05 vs. PTS, ^‡^ *p* < 0.05 vs. TPM”.

As compared with PTS-TPM and PTS-TIC, PTS exhibited the lowest in vitro drug release of 32.99 ± 2.09% at 60 min (Figure 3). It has been suggested that PTS’s poor solubility may be responsible for this. There was a significant increase in the amount of PTS dissolved when both TPM and TIC formulations were used.

The PTS release from TPM at 1 h was approximately 58.95 ± 4.40%. In comparison with pure PTS and TPM, PTS-TIC prepared by microwave irradiation technology produced a considerable improvement in PTS release. After 1 h, 74.03 ± 4.47% of PTS was released from the complex PTS-TIC (Figure 3). In another study, PTS release was reported to be 72% and 87% in 15 min for a binary (PTS: βCD) and a ternary (PTS: βCD: HPMC) inclusion complex, respectively [57]. Moreover, the co-precipitation product of PTS significantly enhanced PTS dissolution in another study. More than 85% of the drug was released after 1 h [16]. In the next step of this study, solid-state characterizations of samples (for PTS, βCD, PLF, and PTS-TIC) were determined.

### 2.3. Differential Scanning Calorimetry (DSC)

The DSC technique has been successfully used for determining inclusion complex formation. A DSC thermogram of the inclusion complex clearly indicates that the guest molecule has melted since the drug does not have an endothermic peak, indicating the inclusion complex is formed.

**Figure 4:**
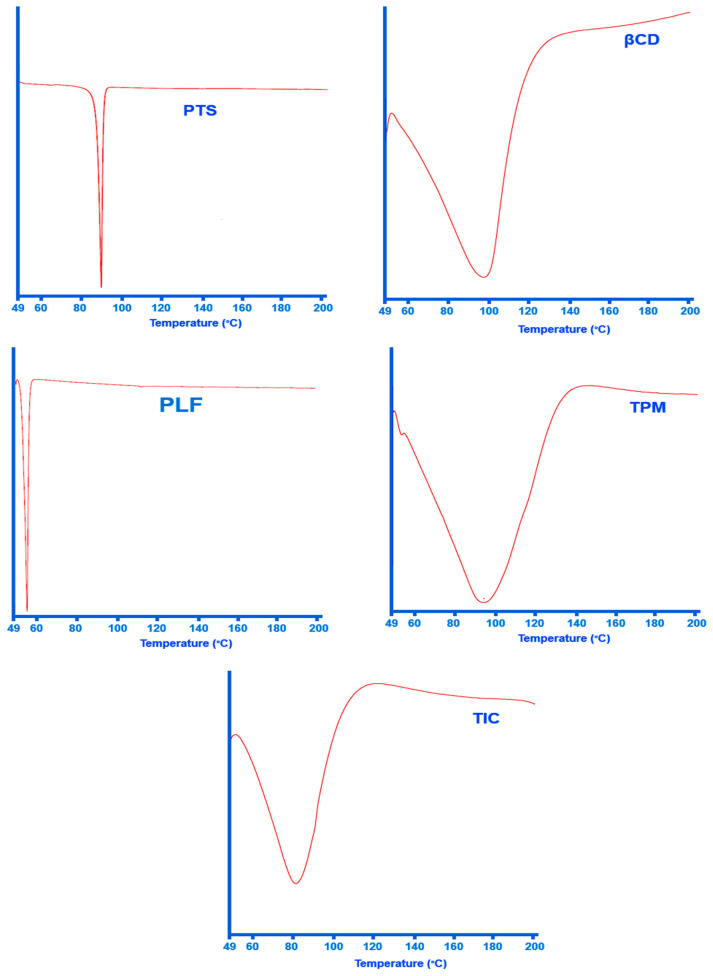
DSC thermograph of PTS, βCD, PLF, TPM, and PTS-TIC.

In accordance with Figure 4 at 95.62 °C, PTS shows a noticeable absorption peak. The absorption peak at 95 °C is attributed to PTS melting, also confirmed by other investigators [16,58]. It is believed that water loss from the βCD structure accounts for the wide endothermic response of the βCD sample at temperatures ranging from 50 °C to 140 °C [56]. In contrast to the PTS DSC curve, no endothermic peak of PTS can be seen at around 95 °C in the PTS-TIC curve. Thus, the vanishing of the endothermic peak related to the drug might be taken as evidence of the formation of ternary complex PTS/βCD/PLF.

### 2.4. Fourier Transform Infrared (FTIR) Spectroscopy

**Figure 5:**
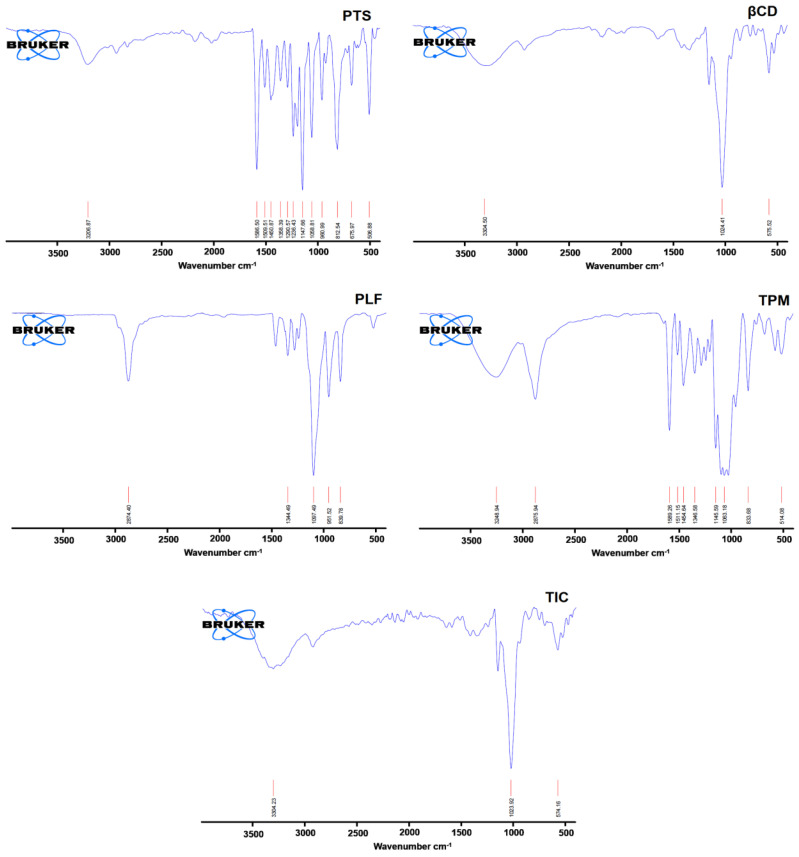
Spectrum of PTS, βCD, PLF, TPM, and PTS-TIC in FTIR.

A typical absorption peak of PTS appeared at 506.88, 675.97, 812.54, 960.99, 1058.81, 1147.81, 1236.43, 1586.50, and 3206.87 cm^−1^. Still, βCD exhibited the main peak at 575.52, 1024.41, and 3304.50 cm^−1^ (Figure 5).

However, the stretching vibration at 839.78, 51.52, 1097.49 cm^−1^, and 2874.40 cm^−1^ are the most notable peak of PLF. In the TPM, drug, βCD, and PLF peaks are available at 514.08, 833.68, 1063.18, 1145.59, 1346.58, 1454.64, 1511.15, 1589.26, 2875.94, 3248.94 cm^−1^. The finding of the FTIR study revealed that there was a notable asymmetry when comparing the FTIR spectra of pure PTS and inclusion complex spectra. Additionally, there were pronounced reductions in signal strength and a wider peak shape in the FTIR spectrum of PTS-TIC, which may have been caused by interactions between PTS, βCD, and PLF, causing a decrease in peak intensities and displacements of peaks. This is illustrated in Figure 5; the prominent peaks of PTS at 506.88, 812.54, 1147.81, and 1586.50 cm^−1^ almost disappeared from the spectrum of the inclusion complex. It was evident that each of these shifts in the spectra revealed PTS incorporation into βCD’s hydrophobic interior.

### 2.5. Nuclear Magnetic Resonance (NMR) Spectroscopy

In this study, a triplet was detected at 2.07 ppm, and a doublet was detected at 6.75 ppm, 6.87 ppm, and 7.46 ppm in the proton NMR spectrum of PTS in acetone-d6. PTS also indicated singlet peaks at 2.08, 3.83, 6.40, 6.99, 7.01, 7.18, and 7.20 ppm.

**Figure 6:**
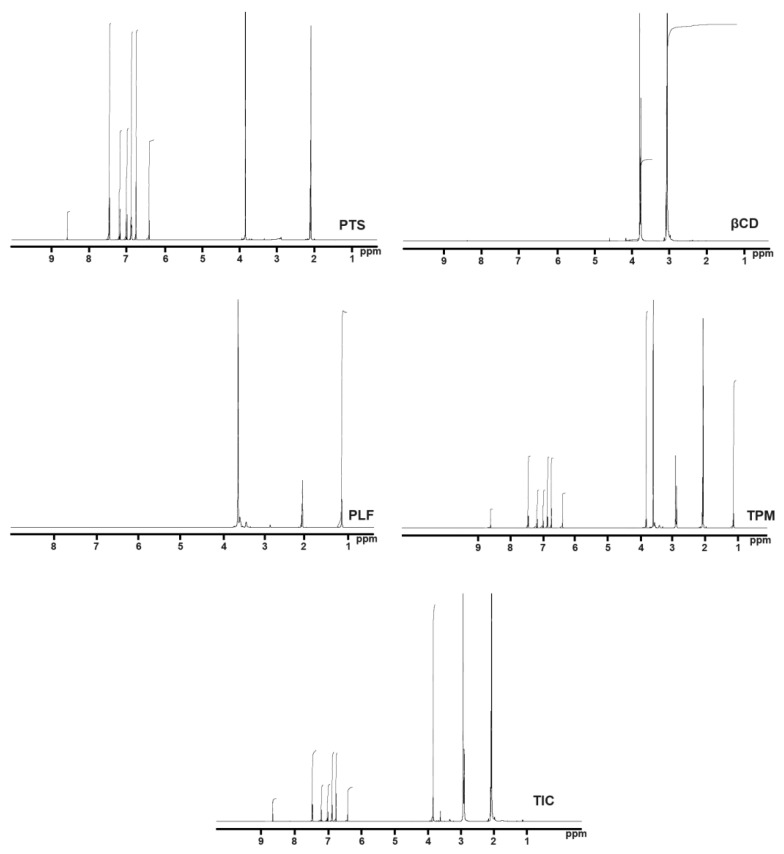
Illustration showing spectrums of PTS, βCD, PLF, TPM, and PTS-TIC determined by NMR.

Meanwhile, βCD molecules presented doublet peaks at δ value of 2.09 and 3.32 ppm (Figure 6). Moreover, there were also distinct singlet peaks found between 1.98 ppm and 2.19 ppm and 2.80 ppm and 2.96 ppm. A triplet peak was observed in the PLF NMR spectrum at 2.07 ppm. As illustrated in Figure 6, there were two distinct peaks at 1.14 ppm and 3.61 ppm. As can be seen from the NMR spectra, the complex PTS-TIC spectrum displays apparent variations in the δ values in comparison with the spectrum of NMR of pure PTS, βCD, and PLF. According to the ^1^H NMR values of PTS-TIC, there was an apparent shift in the βCD and PLF peaks. In the PTS-TIC sample, there were additional peaks related to βCD and PLF, suggesting the formation of TIC. In conclusion, the NMR study implies an interaction between PTS, CD, and PLF that results in inclusion complex development.

### 2.6. Powder X-ray Diffraction (PXRD)

**Figure 7:**
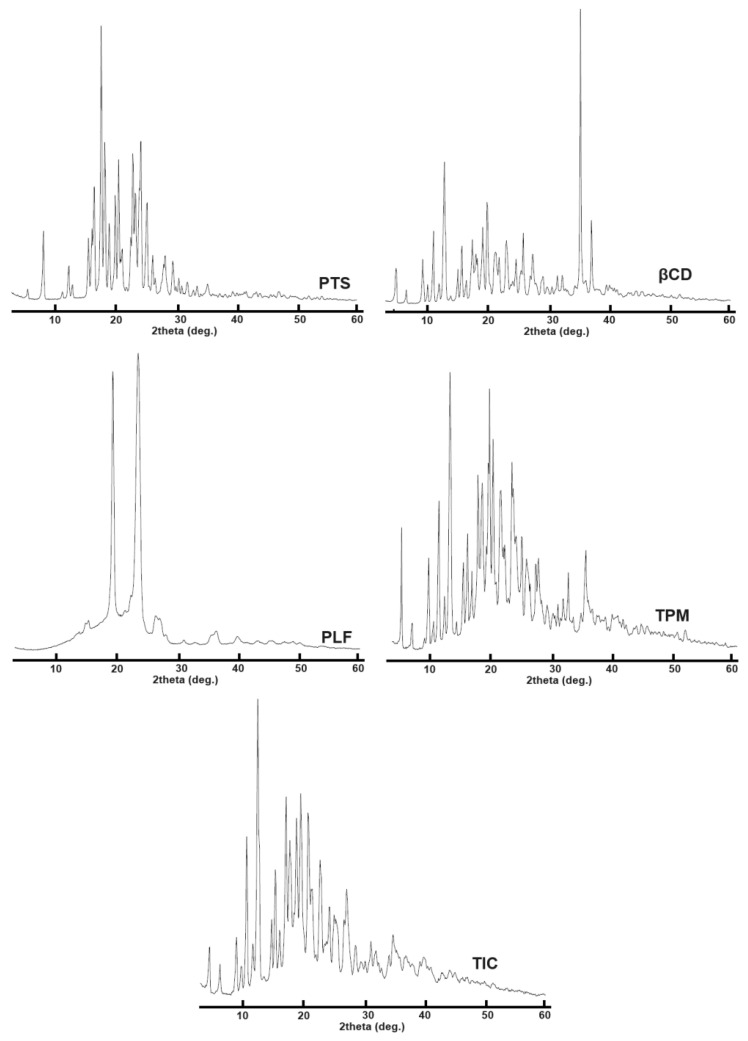
Presented the PXRD plots of PTS, βCD, PLF, TPM, and PTS-TIC.

According to Figure 7, A PXRD image of the PTS sample showed a prominent peak pattern at 2*θ* values of 16.6, 17.4, 18.8, 20.7, 23.0, and 24.3, confirming its crystalline structure.

According to PXRD analysis, βCD exhibits peaks at 2*θ* values of 12.7, 19.1, 23.0, 35.0, and 37.0 (Figure 7). PXRD results for the PTS-TPM indicate that both guest and host molecules were present, thus showing it to be a mixture of PTS/βCD/PLF. Comparing the PXRD patterns, it is observed that the PTS-TIC PRXD pattern is close to the pattern of the βCD PXRD, indicating that the PTS molecule was enclosed in the βCD cavity, which completely concealed the PTS molecule with X-rays. The PXRD analysis of the PTS-TIC does not show distinctive peaks that are typically found in the XRD pattern of PTS. According to the PTS-TIC X-ray diffraction pattern, PTS complexes with the host molecule grid (βCD). The vanishing of crystalline XRD peaks indicates evidence for the occurrence of an inclusion complex [59]. The results of the PXRD analysis are correlated with DSC and FTIR findings.

### 2.7. Scanning Electron Microscopy (SEM)

**Figure 8:**
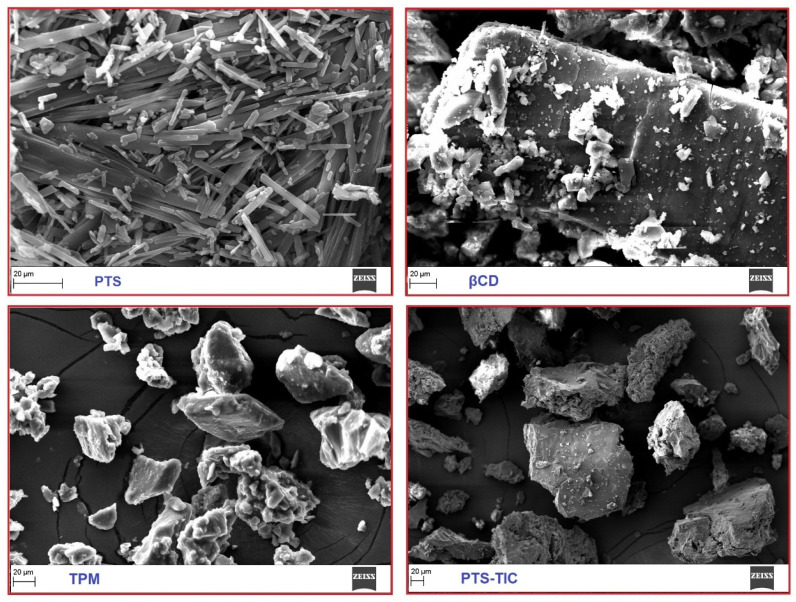
An overview of the SEM images of PTS, βCD, TPM, and PTS-TIC.

In this study, the samples of PTS, βCD, TPM, and TIC were characterized using SEM. The surface of PTS appears to be fairly smooth and compact (Figure 8).

An appearance of thick and rough surface is present in the βCD sample. It is evident in the TIC image that PTS and βCD possess different attributes. TIC complexes did not reflect PTS’s solid, compact appearance. On observing a sample of TIC, PLF seemed to be layered on the surface of PTS/βCD. The morphology and crystal structure of TIC samples strongly support the formation of inclusion complexes.

### 2.8. Antioxidant Activity

**Figure 9:**
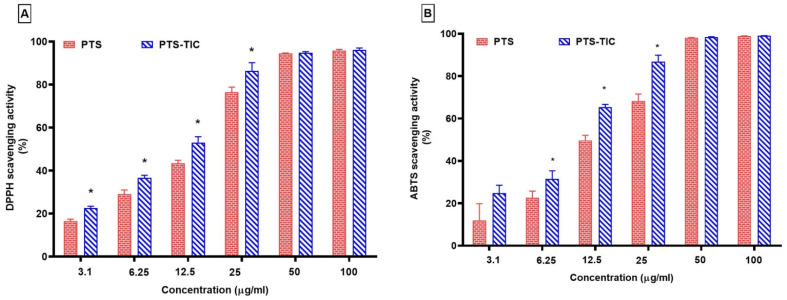
Scavenging of (**A**) DPPH and (**B**) ABTS by PTS and PTS-TIC, “* *p* < 0.05 vs. PTS”.

Our study revealed that PTS and PTS-TIC were both capable of scavenging the DPPH radical significantly when their concentrations were increased. In terms of their DPPH radical scavenging activity, PTS showed 95.86 ± 0.50% and PTS-TIC demonstrated 96.17 ± 0.84% at their maximum concentrations (Figure 9).

PTS-TIC demonstrated a significant increase in DPPH radical scavenging activity up to 25 μg/mL concentration as compared to solo PTS. Also, the PTS-TIC sample exhibited markedly higher levels of ABTS scavenging than PTS up to 25 μg/mL (Figure 9). It was noted that PTS displayed an ABTS radical scavenging activity of 98.90 ± 0.11%, and PTS-TIC showed an ABTS radical scavenging activity of 99.02 ± 0.05% at 100 μg/mL. It was noticed that PTS’s antioxidant activity was clearly impacted by its complexation with βCD and PLF. It was observed that PTS-TIC reacts more quickly with DPPH and ABTS than its free form, with the exception of higher concentrations at 50 μg/mL and 100 μg/mL. This study showed that the prepared inclusion complex PTS-TIC displayed considerable antioxidant properties, potentially due to an increase in PTS solubility when the inclusion complex was prepared under microwave irradiation conditions. It is evident from the findings of the current investigation that CDs are suitable for use as excipients in PTS-containing formulations. The prepared TIC complex resulted in improved PTS physicochemical properties. During this study, PTS’ aqueous solubility improved. In addition, its dissolution rate has been increased, which may lead to an increase in its oral bioavailability. Additionally, PTS-TIC was found to exhibit greater antioxidant activity than PTS alone. Therefore, PTS-TIC could be more effective at preventing oxidative stress and free radical damage.

## 3. Materials and Methods

### 3.1. Materials

The PTS, as well as βCD, were sourced from “Sigma-Aldrich (St. Louis, MO, USA)”. PLF was sourced from Anatrace Products, LLC, Maumee, OH, USA. Potassium dihydrogen phosphate and sodium hydroxide were brought from “Central drug house (P) Ltd. New Delhi, India and Merck, Darmstadt, Germany”, respectively. Ethanol, as well as methanol, came from “Fisher Scientific U.K. Limited Loughborough, UK” and “Scharlab S. L. Sentmenat, Spain” respectively. The rest of the materials were of analytical grade.

### 3.2. Phase Solubility Analysis

A phase solubility study was conducted to evaluate the Ks and Ce of ternary mixtures PTS/βCD/PLF. In the present study, excessive quantities of PTS were added to aqueous solution having different concentrations of βCD from 2 mM to 8 mM and PLF (10%). The samples were placed in water shaker bath for seventy-two hours at 25 °C [60,61,62,63]. After 72 h, the supernatant from each sample was carefully pipetted out and filtered through 0.45 μm membrane filters (“Chromafil^®^Xtra, Macherey-Nagel GmbH & Co. KG, Düren, Germany”). The drug content in the samples was analyzed by UV spectrophotometer at 318 nm [64,65]. According to Equation (1), a slope-based calculation of Ks can be performed using the phase solubility plot [66,67,68,69]. Equation (2) was used to calculate Ce for each sample [66,70,71]. *S*_0_ is the equilibrium solubility of PTS in water [72,73].

(1) $$Ks=\frac{Slope}{Intercept\;(1-Slope)}$$

(2) $$C_{e}=S_{0}Ks$$

### 3.3. Formulation of a Physical Mixture and TIC

For the preparation of a TPM, each ingredient was accurately weighted and carefully blended with all the ingredients of PTS-TPM [PTS: βCD: PLF (10% *w*/*w*)] in a mortar and pestle. The resulting PTS-TPM was preserved in a desiccator for further evaluation. Further, the PTS-TIC was formulated by properly mixing weights of PTS, βCD, and PLF into water containing ethanol. The mixture was then placed in a microwave and irradiated with microwave radiation. The samples were allowed to cool, and cooled samples were collected. A mortar and pestle were used to grind the dried material, and then the prepared sample of PTS-TIC was preserved in an air-sealed container for further evaluation [7,74,75].

### 3.4. Dissolution Study

A USP dissolution apparatus II paddle system (“Sotax, Allschwil, Switzerland”) was used to conduct the dissolution studies. In a dissolution test vessel, samples like PTS, TPM, and TIC were placed for assessment of dissolution. Each vessel contained phosphate buffered solution (pH 6.8, 900 mL) having temperature of 37 ± 0.5 °C [65]. The paddles were rotated at 100 rpm. At each time point up to 60 min, 5 mL of the sample was pipetted out and refilled with the same volume of dissolution vehicle. A UV spectrophotometer at 318 nm was used to measure drug content in the samples [64,65].

### 3.5. Differential Scanning Calorimetry

The DSC system was used in order to determine the thermal properties of PTS, βCD, PLF, PTM, and TIC. An aluminum pan crimped with 5 mg of test sample was loaded in the DSC system (“Perkin Elmer DSC-8000, Waltham, MA, USA”), and testing was conducted using the DSC system at a steady temperature of 10 °C/min. In this experiment, temperature was set between 45 °C and 200 °C.

### 3.6. Fourier Transform Infrared Spectroscopy

Further analysis of PTS, βCD, PLF, PTM, and TIC samples was performed using FTIR spectroscopy “Bruker Alpha FTIR spectrometer (Billerica, MA, USA)” based on the KBr disk approach. After gentle grinding with anhydrous KBr, the samples were compressed into pellets, and samples were scanned over a 400 cm^−1^ to 4000 cm^−1^ spectral range.

### 3.7. Nuclear Magnetic Resonance Spectroscopy

The samples of the PTS, βCD, PLF, PTM as well as the PTS-TIC have been dissolved in acetone-d6. ^1^HNMR scans were carried out by “Bruker NMR spectroscopy”. Spectral and chemical shift measurements are displayed as ppm.

### 3.8. Powder X-ray Diffraction (PXRD)

X-ray diffractometer measurements were performed on the PTS, βCD, PLF, TPM, and inclusion complex to determine its physical state. Analyses were performed on all test samples by “Ultima IV Diffractometer” using a 2-theta range of 3°–60°.

### 3.9. Scanning Electron Microscopy

The features of the surface of PTS, βCD, PLF, TPM, and PTS-TIC were characterized using an “EVO LS10 microscope (Carl Zeiss, Oberkochen, Germany)”. The samples were scanned individually by SEM, and photographs were electronically captured under magnification.

### 3.10. DPPH Scavenging Activity and ABTS Radical Cation Scavenging Activity

The antioxidant activity of prepared PTS-TIC was estimated by the DPPH assay. A concentration range of 0–100 g/mL was prepared by dissolving PTS and PTS-TIC separately in methanol. The absorbance at 517 nm was recorded by spectrophotometer [73]. Samples were calculated based on their ability to scavenge DPPH free radicals by using Equation (3).

An analysis of the radical scavenging activity of PTS-TIC against ABTS radical cations was conducted [76]. Potassium persulfate and ABTS solutions were prepared separately in water at a concentration of 2.45 mmol/L and 7 mmol/L, respectively. In this study, A 1:1 mixture of both solutions was prepared and allowed to remain at room temperature for 6 h in a dark compartment; ABTS radicals were produced during this period. In the next step, mixing diluted ABTS radical cation solution (2.9 mL) with 0.1 mL of PTS-TIC was performed. Analyses were carried out at 734 nm after incubating the reaction for 20 min at 30 °C. In accordance with Equation (3), the test sample’s efficiency in quenching the ABTS free radical was assessed.

(3) $$Scavenging\;activity\;\left(\%\right)=\frac{A_{control}-A_{test}}{A_{control}}\times 100$$

### 3.11. Statistical Analysis

“Statistical analysis of dissolution profile was assessed by one-way ANOVA followed by Tukey’s test. Statistical analysis of phase solubility and antioxidant activity was carried out using the unpaired *t*-test. All analysis was conducted with GraphPad InStat^®^ (https://www.graphpad.com/scientific-software/instat/). The significant difference was determined by *p* < 0.05”.

## 4. Conclusions

In this study, microwave irradiation was used to prepare PTS ternary inclusion complexes with βCD/PLF. The findings from the phase solubility study suggested that βCD/PLF enhanced the solubility of PTS. In this study, it was found that PTS displayed greater solubility in the presence of βCD/PLF. Further, it was found that 74% of PTS was released from the prepared complex PTS-TIC at 60 min. A significant enhancement in the PTS dissolution was observed in the ternary complex (*p* < 0.05) over the TPM. This could be due to the presence of PLF, which may contribute to the enhancement of the solubility of PTS. There has been confirmation of ternary inclusion complex formation based on solid-state characterization tests. Moreover, the PTS/βCD/PLF ternary inclusion complex substantially augmented the PTS antioxidant activity up to 25 μg/mL, possibly due to an increase in PTS solubility during the preparation of the inclusion complex. According to these results, the preparation of inclusion complexes having a CD with poorly soluble pharmacological agent(s) may be an efficient strategy to facilitate the solubility and dissolution performance of the active(s).

## Data Availability

Data are contained within the article.
